# G-Quadruplex Forming DNA Sequence Context Is Enriched around Points of Somatic Mutations in a Subset of Multiple Myeloma Patients

**DOI:** 10.3390/ijms25105269

**Published:** 2024-05-12

**Authors:** Anna S. Zhuk, Elena I. Stepchenkova, Irina V. Zotova, Olesya B. Belopolskaya, Youri I. Pavlov, Ivan I. Kostroma, Sergey V. Gritsaev, Anna Y. Aksenova

**Affiliations:** 1 Laboratory of Amyloid Biology, St. Petersburg State University, 199034 St. Petersburg, Russia; aszhuk@itmo.ru (A.S.Z.); info@grayhawk.spb.ru (I.V.Z.); 2 Institute of Applied Computer Science, ITMO University, 197101 St. Petersburg, Russia; 3 Vavilov Institute of General Genetics, St. Petersburg Branch, Russian Academy of Sciences, 199034 St. Petersburg, Russia; stepchenkova@gmail.com; 4 Department of Genetics and Biotechnology, St. Petersburg State University, 199034 St. Petersburg, Russia; 5 Resource Center “Bio-Bank Center”, Research Park of St. Petersburg State University, 198504 St. Petersburg, Russia; olesya.belopolskaya@gmail.com; 6 The Laboratory of Genogeography, Vavilov Institute of General Genetics, Russian Academy of Sciences, 119991 Moscow, Russia; 7 Eppley Institute for Research in Cancer, Fred and Pamela Buffett Cancer Center, University of Nebraska Medical Center, Omaha, NE 68198, USA; ypavlov@unmc.edu; 8 Departments of Biochemistry and Molecular Biology, Microbiology and Pathology, Genetics Cell Biology and Anatomy, University of Nebraska Medical Center, Omaha, NE 68198, USA; 9 City Hospital No. 15, 198205 St. Petersburg, Russia; obex@rambler.ru (I.I.K.); gritsaevsv@mail.ru (S.V.G.)

**Keywords:** G-quadruplex structures, multiple myeloma, Next Generation Sequencing (NGS), somatic mutations, mutational signatures

## Abstract

**Simple Summary:**

Genomic instability is an important feature of cancer, including multiple myeloma, which is the second most common hematological malignancy. There are several sources of genomic instability in multiple myeloma, including mutations in DNA repair genes and genotoxic therapy. Non-canonical secondary DNA structures (such as four-stranded G-quadruplex structures) may contribute to this process by interfering with DNA replication and repair and leading to the accumulation of mutations at specific sites in the genome. Here, we address the question of whether G-quadruplex structures have any impact on the accumulation of mutations in multiple myeloma cells. We discuss the possible consequences of defects in G-quadruplex unwinding for the specificity of somatic mutations in MM. Understanding the role of G-quadruplex structures in the disease may lead to the development of new diagnostic and therapeutic strategies for multiple myeloma and other cancers.

**Abstract:**

Multiple myeloma (MM) is the second most common hematological malignancy, which remains incurable despite recent advances in treatment strategies. Like other forms of cancer, MM is characterized by genomic instability, caused by defects in DNA repair. Along with mutations in DNA repair genes and genotoxic drugs used to treat MM, non-canonical secondary DNA structures (four-stranded G-quadruplex structures) can affect accumulation of somatic mutations and chromosomal abnormalities in the tumor cells of MM patients. Here, we tested the hypothesis that G-quadruplex structures may influence the distribution of somatic mutations in the tumor cells of MM patients. We sequenced exomes of normal and tumor cells of 11 MM patients and analyzed the data for the presence of G4 context around points of somatic mutations. To identify molecular mechanisms that could affect mutational profile of tumors, we also analyzed mutational signatures in tumor cells as well as germline mutations for the presence of specific SNPs in DNA repair genes or in genes regulating G-quadruplex unwinding. In several patients, we found that sites of somatic mutations are frequently located in regions with G4 context. This pattern correlated with specific germline variants found in these patients. We discuss the possible implications of these variants for mutation accumulation and specificity in MM and propose that the extent of G4 context enrichment around somatic mutation sites may be a novel metric characterizing mutational processes in tumors.

## 1. Introduction

Multiple myeloma (MM) is a malignant neoplasm of terminally differentiated immunoglobulin-producing B lymphocytes called plasma cells. MM is the second most common hematologic malignancy, and it poses a heavy economic and social burden. MM is characterized by high genetic heterogeneity. The genomes of tumor cells in patients with MM carry numerous structural variations, chromosomal gains and losses, and point mutations affecting different cellular pathways, including genome maintenance. For a comprehensive review of the processes leading to genome destabilization in MM, see [1]. Among the many factors that form the specific mutational profile of MM, the role of non-canonical four-stranded G-quadruplex structures of DNA (G4) deserves special attention, due to the relatively limited number of studies on the subject.

G-quadruplexes are four-stranded structures in nucleic acids which are formed through Hoogsteen base pairing between four guanines in planar tetrads and stabilized by π–π–stacking interactions between these G-quartets [2,3,4,5]. The number of stacked G-quartets defines the stability of the whole structure with 3 or more G-quartets being thermodynamically highly stable. G-quadruplex structures are highly polymorphic and are classified based on several factors, such as orientation of the strands (parallel, antiparallel, or hybrid), glycosidic conformation of guanines (syn- or anti-), and loop connectivity (edgewise, diagonal, double-chain-reversal or V-shaped loops). The formation of G-quadruplexes can involve one molecule (intramolecular G-quadruplexes) or several molecules (intermolecular G-quadruplexes). G-quadruplexes are naturally formed in genomic DNA, where they play a role in processes such as gene expression regulation, chromosome organization, and chromosome end protection [6]. G-quadruplexes are abundant in regulatory sequences in genes (promoters and enhancers), at telomeres, and at recombination sites [7,8,9].

The formation of G-quadruplexes in vivo was visualized by immunostaining with specific high-affinity single-chain antibodies or by fluorescent probes and has been mapped in different regions of genomic DNA in various species including ciliates and humans [10,11,12,13,14,15]. High-throughput G4-seq of human genome allowed to build a high-resolution map of G4s and showed that their formation was significantly associated with oncogenes, tumor suppressors, and somatic copy number alterations related to cancer development [16]. G-quadruplex structures occurred more frequently in the nuclei of cancer cells compared to the corresponding non-neoplastic tissues [17]. The association of G-quadruplexes with oncogene promoters prompted the investigation of various G4-ligands as anticancer agents [18,19,20,21].

Multiple studies suggest that G4 structures play an essential regulatory role in the genome [22]. Thus, G4s in promoters are associated with high transcription levels in open chromatin [23,24]. G4s are required for replication initiation [25,26] and high-order chromatin organization [27,28]. In B-cell lineage, G-quadruplexes may form at the IGH locus at (V) variable regions and switch-regions, thereby promoting hypermutation and class-switch recombination [29]. Several studies provide evidence for the association of G-quadruplexes with DNA modifications and function of epigenome [30,31,32]. G4s are highly abundant in human embryonic stem cells and this abundance is lost during cell differentiation [33]. G4s in RNA can regulate alternative splicing and translation [34,35,36].

It is well acknowledged that G-quadruplexes can pose a significant threat to genomic stability [37]. Dealing with such sequences can be challenging for cellular machineries, especially for DNA replication, which can be blocked by such structures [13,38]. G4 structures can form when the DNA double-helix is unwound and is not protected by specific proteins. The unwinding of G4-structures in DNA and RNA requires specialized enzymes capable of dealing with such structures. Several helicases are known to be unwinding G4 structures, including the RecD-homologues Pif1 and Rrm3, RecQ-like enzymes (BLM, WRN) and the Fe-S helicases RTEL1 and FANCJ [39,40].

These helicases are essential guardians of genome stability, and mutations in the corresponding genes are associated with genetic disorders characterized by increased rates of cancer development and premature aging [41,42,43]. G4 structures are amenable to DNA damage and block efficient DNA repair. At the same time, G4s can modulate the activity and function of repair pathways. For instance, they differently regulate the activity of nucleotide excision repair, base excision repair, homologous DNA repair, and non-homologous end-joining [44]. Also, G4 structures can modulate the activity of the DNA mismatch repair system [45,46].

Importantly, non-canonical DNA configurations, including G4s, are among the major factors driving the accumulation of somatic mutations in cancer cells [47,48]. Translocation breakpoints were enriched at sequences with the potential to form G4 DNA structures in tumor samples that were characterized by elevated genetic instability and frequent mutations in tumor suppressor genes, such as *TP53* [48,49]. Mutations that modulate the stability of G4 in non-coding regions (5′UTR) have been described in cancer genomes [50]. Recently, an association between G4s and somatic structural variants in cancers has also been described [51].

In this study, we examined the G4 context around the mutation sites in multiple myeloma and found enrichment for G4 motif percentage in tumors from several patients. We analyzed the mutational signatures in these tumors and their association with the groups classified by the G4 context enrichment. In addition, we studied germline mutations carried by the patients and found variants in the genes encoding for the DNA repair components that are characteristic of the patients with enrichment of somatic mutations around G4 contexts. We propose that G4 context enrichment around somatic mutation sites can characterize mutational processes in tumors and discuss possible implications of the defects for DNA repair and G4 unwinding for somatic mutations specificity in MM.

## 2. Results

### 2.1. Analysis of the Mutation Context around Tumor Mutation Sites

Analysis of somatic mutational patterns is a powerful tool for understanding the etiology of human cancers [52,53]. Different mutational processes operating in cancer genomes may generate characteristic mutational signatures or patterns distinguishing different tumors and providing the background for tumor variability and evolution.

We analyzed exome NGS data obtained from normal and tumor samples of 11 patients who were newly diagnosed with multiple myeloma. The characteristics of patients are provided in Table 1.

**Figure 1:**
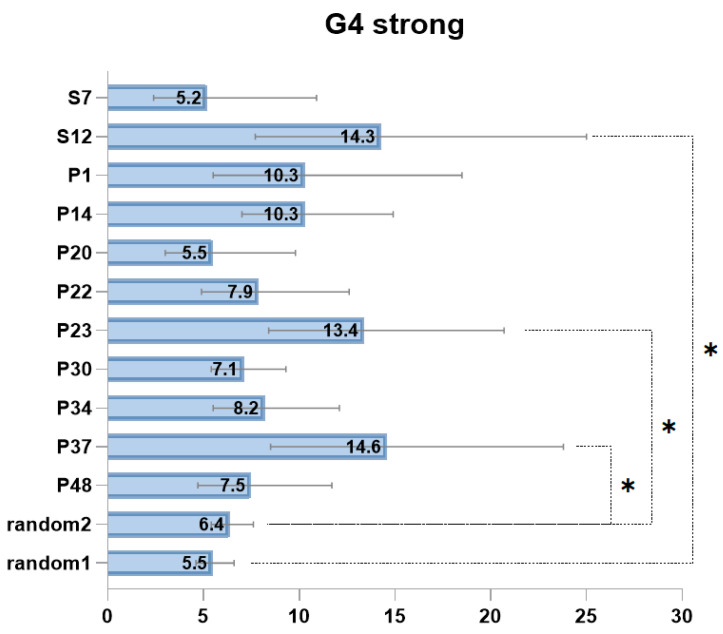
Percentage of G4 strong context occurrence near mutation sites in different patients and in randomly sampled sequences. The random1 and random2 sets include 2000 randomly selected sequences from genomic intervals corresponding to the All Exon V6+UTR V6 enrichment panel (random2) or Truseq Exome panel (random1). The graph displays the percentage proportion along with the confidence interval for the proportion. The asterisk denotes a statistically significant difference between the proportions of G4 context occurrence around point of somatic mutations in patients and in randomly sampled sequences as determined by a z-test.

Since there are accumulating data on the role of G4 structures in somatic genome changes in cancer, we decided to analyze G4 context in the vicinity of somatic mutations in patients with MM. First, we extracted sequences of 70 nucleotides up- and downstream of the somatic mutation sites found in the tumor genome, and second, we analyzed them for G4 context. We searched for G4 weak and G4 strong contexts as described in the Materials and Methods section. As a control, we used a randomly generated set of sequences from the same exome regions and determined the number of sequences containing G4-forming motifs. Overall, we found enrichment in G4 strong context in tumors from 3 patients when compared to the randomly generated set (Table 2, Figure 1). In tumor from patient P48, we found a significant enrichment for the combined G4 weak and G4 strong context due to the high percentage of somatic mutations in the predicted G4 weak regions (Table 2).

As seen in Table 2, patients with G4 strong context enrichment carried fewer mutations in their tumors compared to patients without G4 enrichment (88 vs. 245.6, on average per tumor). According to this analysis, all tumors were further classified into two groups: (i) enriched with G4 strong context around mutation sites and (ii) without G4 strong context enrichment.

### 2.2. Mutational Signatures Found in the Tumors of the Patients Studied

**Figure 2:**
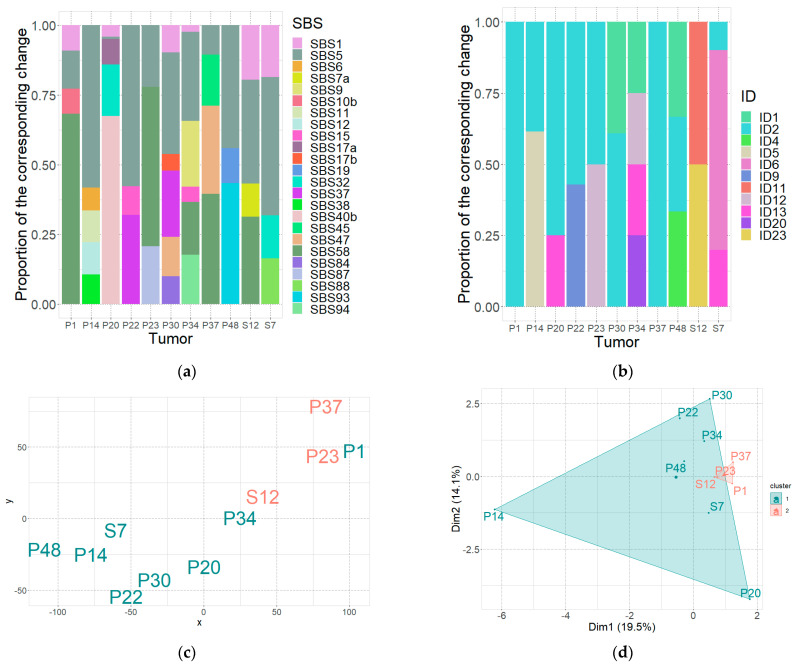
Mutation signatures observed in the analyzed tumors. (**a**) Visualization of SBS proportions in each of the analyzed tumors based on SigProfilerAssignment. (**b**) Visualization of small insertions and deletions (ID) among somatic mutations determined in different patients by SigProfilerAssignment. (**c**) t-SNE analysis based on SigProfilerAssignment SBS classification, percentage of SBS in each sample used, samples with G4 strong context enrichment are salmon, samples without G4 strong context enrichment are cyan. (**d**) k-means cluster analysis based on SigProfilerAssignment SBS classification was performed for illustration of similarity between samples; percentage of SBS in each sample used. Mutational signature associations: SBS1—aging, clock-like signature, spontaneous or enzymatic deamination of 5-methylcytosine to thymine; SBS5—aging, clock-like signature, may implicate NER [55]; SBS6—defective DNA mismatch repair, is very specific to MM with high genomic risk [56]; SBS7a—DNA damage due to exposure to ultraviolet light; SBS9—activity of activation-induced deaminase (AID) in non-coding regions, mutation pattern found in B-cell cancers that develop after the germinal center stage. This signature results from the off-target activity of AID (normally working during the germinal center phase of the hypermutation of immunoglobulin genes [57], MMR, and gap repair with participation of DNA polymerase eta); SBS10b—polymerase epsilon exonuclease (POLE-Exo) domain mutations [58]; SBS11—a mutation pattern similar to that of alkylating agents; SBS12—defective mismatch repair [59]; SBS15—defective DNA mismatch repair [60]; SBS17a and b—unidentified etiology, were found in MM [61]; SBS32—treatment with azathioprine prior to induce immunosuppression, the presence of transcription-coupled nucleotide excision repair activity on damaged DNA [62]; SBS38—indicating possible secondary harm caused by UV exposure; SBS40b—related to indicators of decreased kidney function; SBS84—activity of AID [62,63]; SBS87—thiopurine chemotherapy treatment; SBS88—explore to the colibactin from *E. coli*-carrying pks pathogenicity island, displays heightened activity during early childhood; SBS19, SBS37, SBS93, SBS94—unknown; SBS45, SBS47, SBS58—possible sequencing artefact. ID1, ID2—indicate DNA mismatch repair deficiency; ID5—possible clock-like signature; ID6—defective homologous recombination repair; ID13—UV exposure; ID23—aristolochic acid exposure; ID4, ID9, ID11, ID12, ID20—unknown.

To further assess the differences in mutational processes between two groups of patients, we analyzed mutational signatures for single base substitutions (SBSs) and indels (IDs) in each tumor using SigProfilerAssignment [54]. We found that mutation signatures varied significantly in the analyzed tumors (Figure 2a,b). Among the most frequently occurring SBS signatures were SBS1 (6/11 tumors) and SBS5 (11/11 tumors). The SBS1 signature is proposed to be caused by spontaneous or enzymatic deamination of 5-methylcytosine to thymine, while SBS5 has an unknown etiology. Among the indels, ID2, ID1, and ID13 were the most frequently observed. It is known that ID1 and ID2 signatures typically account for 45% of indels in non-hypermutated cancer genomes [52].

Next, we asked whether tumors from the group with G4 strong enrichment carried some specific mutational signatures that could allow them to differentiate this group from the second group. For visual interpretation of the SBS mutational signatures in different samples, we performed t-SNE algorithm and k-means cluster analysis on the data obtained from the SigProfilerAssignment mutational signature classification. As seen in Figure 2c,d, the samples belonging to the group with G4 strong enrichment separate from the other samples and cluster together. All of these samples were characterized as carrying SBS58 mutational signature (see Figure 2a). The SBS58 signature is characterized mostly by C→T and T→C changes in the W-context from 3′ and 5′ ends and has an unknown etiology, sometimes attributed to sequencing artefacts. Interestingly, this signature shows transcriptional strand asymmetry (https://cancer.sanger.ac.uk/signatures/sbs/sbs58/, accessed on 21 February 2024), which is also typical of mutagenesis in G4-forming regions.

**Figure 3:**
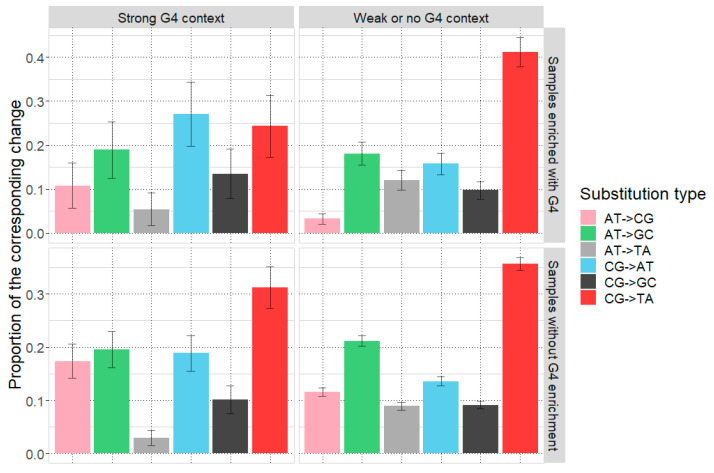
Types of mutations in samples with G4 context enrichment around points of somatic mutations and without G4 context enrichment, classified by the type of context. Standard deviation of a proportion is shown as error bars.

In addition to this analysis, we studied types of base substitutions in samples from the two analyzed groups (with G4 strong enrichment and without) classified by the presence or absence of the G4 strong context around mutation sites (see Figure 3). C→A and A→C mutations were elevated specifically in regions with G4 strong context in samples enriched with G4 strong context, while C→T mutations were decreased.

### 2.3. Classification of Somatic Mutations according to the Type of Substitutions and Their Predicted Consequence

**Figure 4:**
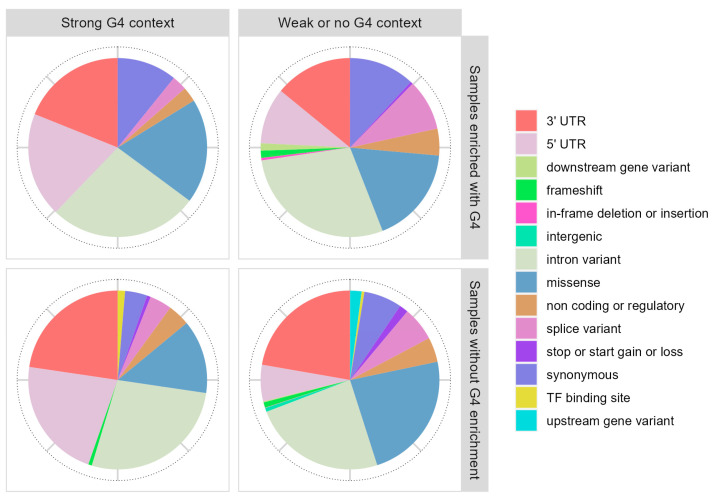
Consequence of somatic mutations found in different groups of samples in respect to the G4 context.

Mutations in the G4 context are more frequently found in the upstream and downstream regions of the genes such as 5′ and 3′UTRs, where G4 structures are more frequently observed and might have a regulatory role (Figure 4).

As known from the literature, somatic mutations may modulate the stability of G4 in non-coding regions in cancer genomes, which may affect gene expression [50]. We screened for predicted structural changes in the analyzed regions considering somatic mutations found and detected 33 cases in total when somatic mutations changed the prediction of the G4-forming properties of the analyzed region (see Figure S1). The majority of these changes were detected in 5′ and 3′UTRs, introns, and coding regions (see Figure S2). Whether these changes may lead to changes in expression of the corresponding genes needs further investigation.

### 2.4. Germline Variants Found in Patients

**Figure 5:**
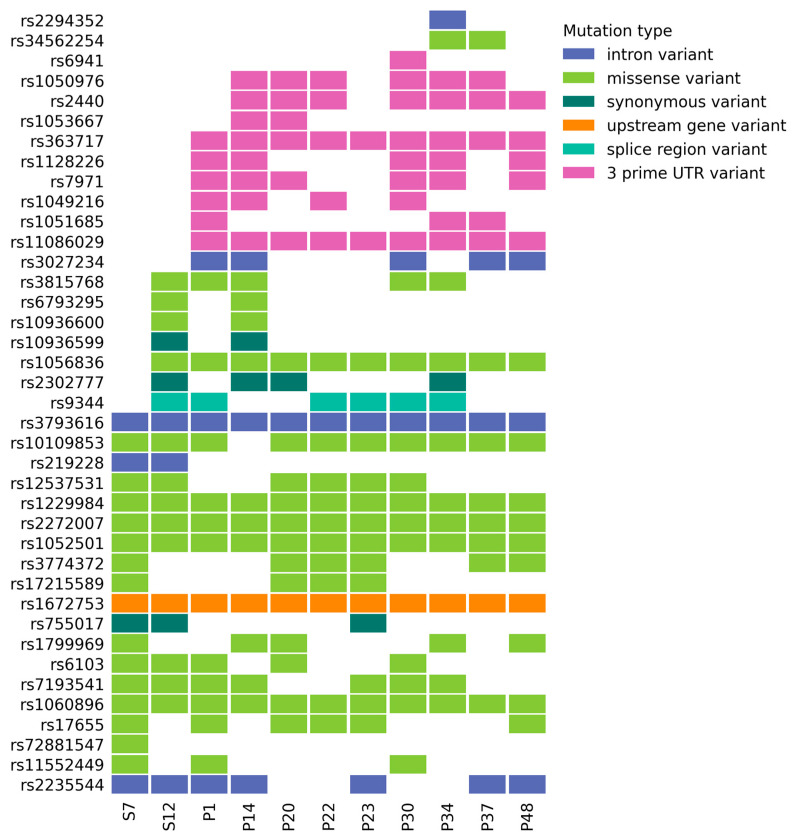
Germline SNPs associated with MM according to publications, GWAS catalog, and Clinvar. SNPs affect such genes as *XRCC5*, *ULK4*, *ADH1B*, *ELL2*, *NDUFA8*, *CCND1*, *SLC28A2*, *RFWD3*, *CTC1*, *TNFRSF13B*, *KLF2*, *ZBTB46*, *MYNN*, *LRRC34*, *SMARCD3*, *ICAM1*, *SAA4*, *DCLRE1B*, *CASP3,* and *MRTFA*. CoMut were used for SNP visualization [64].

We wondered whether the patients with enriched G4 strong context at the mutation sites carried specific SNPs associated with multiple myeloma predisposition. For this purpose, we analyzed germline SNPs known to be associated with multiple myeloma. We did not detect a significant difference between the groups of patients with and without the G4 enrichment. Patients carried known SNP variants in the genes *XRCC5*, *ULK4*, *ADH1B*, *ELL2*, *NDUFA8*, *CCND1*, *SLC28A2*, *RFWD3*, *CTC1*, *TNFRSF13B*, *KLF2*, *ZBTB46*, *MYNN*, *LRRC34*, and *SMARCD3*, whereas the variants rs1799969 (*ICAM1*), rs72881547 (*SAA4*), rs11552449 (*DCLRE1B*), rs1049216 (*CASP3*), and rs2294352 (*MRTFA*) were found only in patients without G4 strong enrichment pattern (see Figure 5). Additionally, we analyzed germinal variants in genes encoding components of DNA repair machinery and associated proteins (see Figure S3). Samples from patients S12, P23, P37, and P48, where we found enrichment in G4 structure prediction, carried germinal variants in the *LARP7* gene, distinguishing them from the other samples (see Section 2.5 for more details).

### 2.5. Identification of the Germline SNPs Common to the Patients with the G4 Strong Context Enrichment in Tumors

Next, among all SNPs detected in patients, we searched for SNPs common to the G4 strong group and absent in all other patients. In total, we found 15 SNPs in 14 genes common to the G4 strong group (Tables S1 and S2). Eight of these SNPs have a relatively low population frequency (below 0.1), which is not in favor of their random appearance in all patients of the group. These SNPs affect several genes that are related to DNA repair, chromatin modification, and cancer. One of these genes is *LARP7*, encoding a La family RNA-binding protein. The identified missense variant (rs79383654, the minor allele A) in *LARP7* results in E4K change at the very N-terminus of the protein that is predicted to be disordered. Importantly, LARP7 is a BRCA1 ubiquitinase substrate involved in homology-directed repair (HDR), and its deficiency attenuates DNA damage response (DDR) [65]. LARP7 has also been shown to activate the SIRT1 deacetylase and prevent DDR-induced cellular senescence [66]. Along with its interacting partner MEPCE, LARP7 is involved in the release of stalled RNA polymerase II (RNAPII), and their depletion in BRCA1-deficient cells leads to R-loop accumulation and replication stress [67,68,69]. *LARP7* is a potential tumor suppressor in gastric and breast cancer [70,71]. It should be noted that patients S12, P23, P37, and P48 (combined G4 strong and G4 weak group) carried another missense-variant rs62317770 in the *LARP7* gene, causing Arg279Gln change in the protein. Altogether, these germinal variants in the *LARP7* gene distinguished them from the other samples (Figure S3).

Another SNP, rs11250255, minor allele T, affects a non-coding region of the *WDR37* gene. The function of WDR37 is currently unknown; however, this protein is known to contain WD40 repeat (WD) domains, representing a common protein interaction domain in humans, generally mediating interactions with other proteins. Missense variants in WDR37 cause a severe multisystemic syndrome in humans [72,73]. WDR37 interacts with PACS1 and PACS2, the multifunctional proteins involved in protein trafficking and DNA repair [73]. Loss of Pacs1 or Wdr37 in mice induces oxidative stress, impairs ER Ca^2+^ efflux in B and T cells after antigen receptor stimulation, and decreases lymphocyte quiescence [74]. Interestingly, PACS1 plays a critical role in chromatin maintenance and genome integrity by mediating the stability of HDAC2 and HDAC3; its deficiency induces genomic instability and replication stress [75]. Upregulation of PACS1 leads to suppression of DDR and development of chemo-resistant tumors [76]. rs3098238, minor allele C, is a synonymous change in the *DCAF13* gene, encoding DDB1-and CUL4-associated factor 13. DCAF13 is a substrate receptor for the cullin RING-finger ubiquitin ligase 4 (CRL4) E3 ubiquitin ligase, which regulates cell cycle progression [77]. DCAF13 is currently viewed as an oncogene [77,78,79,80]. CRL4^DCAF13^ regulates histone H3 lysine-9 methylation and SUV39H1 polyubiquitination and degradation [81]. We have also found that patients in the G4 strong group carried several SNPs affecting the *DDX5* gene, encoding the G4 helicase, which were absent in other patients (Table S3).

## 3. Discussion

Cancer cells accumulate different mutations that can affect tumor growth, cell fitness, genome stability, and mutation accumulation or be neutral. The concept of mutational signatures, introduced in 2012, represents generic patterns of mutations arising during tumorigenesis and depending on endogenous and/or exogenous factors [53,82]. The conceptual development of mutational signatures started from single-base substitution patterns and evolved into more complex patterns, such as those represented by double-base and insertion or deletion (indel or IDs) contexts and finally to structural rearrangement contexts [52,83,84,85,86].

The occurrence of mutations in one or another genome site depends on many factors. One of the most important factors is the structural properties of DNA. It is well known that secondary DNA structures may affect replication and/or transcription, as well as influence repair of DNA damage. If not properly processed, G-quadruplex structures pose a serious threat to genome stability [44]. G-quadruplexes are known roadblocks for DNA replication [39,43]. The DNA replication machinery stalling at G4 structures can lead to replication stress, which is a significant source of genomic instability and somatic mutations [87]. DNA replication across G4 structures usually requires the action of structure-specific helicases. Mutations in the genes encoding various G4-helicases have been associated with inheritable genetic diseases such as Bloom and Werner syndromes, Fanconi anemia, and predisposition to cancer [9]. G4s are important regulatory elements in the genome. For instance, they are frequently observed in or near oncogene promoters, and modulation of G4 formation by specific ligands has been proposed as a powerful tool to treat cancer through the control of oncogene expression [21,88]. G4 motifs in the *TERT* promoter region in primates have shown higher frequency of nucleotide substitutions as compared to the surrounding regions [89]. In diffuse large B-cell lymphoma, AID mutation hotspots were highly enriched for G4 elements, and G4s are thought to be involved in the recruitment of AID to targeted regions within B-cells [90]. G4s can affect the binding affinity and functional responses of MMR proteins [45].

We observed enrichment for the G4 strong sequence context around somatic mutation sites in tumors obtained from patients with multiple myeloma. G4 enrichment was characteristic only for some tumors and was not observed in others, suggesting that a specific genetic or epigenetic background may be responsible for the occurrence of mutations in this context. This does not contradict the data on the high genetic heterogeneity of tumor cells in patients with MM. In one patient, we observed enrichment for the G4 weak context around sites of somatic tumor mutations. These data highlight the heterogeneity of mutational processes occurring in different tumors of the same type. Importantly, the observed difference in the G4 mutational signature may depend on the mutations carried by the patients. We searched across all the germline SNPs detected in the analyzed patients and separated a group of SNPs that are characteristic only for the group with a G4 strong context. Among 15 identified SNPs, we selected a group of eight with a relatively low population frequency, which minimized the possibility of their accidental occurrence in this group. Three of these identified SNPs affected genes *LARP7*, *WDR37,* and *DCAF13*, which are involved in DNA repair and DNA damage response and are associated with carcinogenesis. Importantly, depletion of LARP7 caused R-loop accumulation and promoted replication stress [67,68,69]. This makes the missense mutation rs79383654 (Glu4Lys), affecting *LARP7*, a likely candidate factor influencing the enrichment of G4 strong context around the points of somatic mutations in tumor cells of patients with MM. This variant affects the very N-terminus of the LARP7 protein representing the intrinsically disordered region of the protein [91]. The limitation of our study is the small number of samples analyzed. Further studies may help to understand the significance of rs79383654 in *LARP7* function and its role in mutation accumulation in G-quadruplex-forming sequences.

A significant association between specific mutational signatures and MM subgroups has been previously reported [56,92]. The presence of SBS1 was found to be more prevalent in the hyperdiploid MM subgroup [92]. SBS1 and SBS5 were highly specific for standard risk MM. Signatures SBS3 and SBS6 were particularly targeted towards MM with high-risk genomic rearrangements, and SBS3 was characteristic of functional high-risk groups [56]. We have found that tumors characterized by G4 strong context enrichment are more similar to each other than to the rest of the tumors when different SBSs were analyzed. SBS58 was found in all tumors from the G4 strong enriched group. Although SBS58 often classified as a potential artefact signature, it was described to be elevated in late stage metastatic melanoma samples [93], uveal melanoma [94], and breast cancer [95]. The transcriptional strand bias of this signature and its detection in the G4 enriched group in our experiments may reinforce further studies.

Summing up our observations, the percentage of G4 context enrichment around somatic mutation sites can represent a novel metric describing tumor heterogeneity that may be linked to specific mutational signatures and mutational processes undergoing in different tumors. Expanding patient cohorts and functional validation experiments can bring more information about the mechanisms underlying this phenomenon. Remarkably, groups of patients with different G4 enrichment percentages may respond differently to treatment, and future studies can help explore novel therapeutic implications by targeting these structures or specific mutational processes. Furthermore, the differential G4 enrichment could serve as a biomarker to customize treatment plans, optimize therapeutic outcomes, and predict patient response to specific drugs.

## 4. Materials and Methods

### 4.1. Patients

The study included 11 patients newly diagnosed with MM at the Russian Research Institute of Hematology and Transfusiology, and the City Hospital No. 15, St. Petersburg, Russia (Table 1). Of the study participants, 6 (54.5%) were female and 5 (45.5%) were male. The age of the patients ranged from 56 to 83 years, with a median age of 71 years. The initial somatic status of most patients was satisfactory and ranged from ECOG 1–2, while the somatic status of 3 patients was ECOG 3. All patients had an intermediate comorbidity index (1–2 points) or lower. All patients signed the informed consent in accordance with the Declaration of Helsinki. The study was approved by the Ethics Committee of the Russian Research Institute of Hematology and Transfusiology (St. Petersburg, Russia).

### 4.2. Sequencing of the DNA from Tumor and Normal Samples

After completing the diagnostic procedures and confirming the diagnosis of MM, bone marrow samples of 1–5 mL and blood samples of 10 mL were collected from all pa-tients. CD138+ plasma cells were isolated from bone marrow aspirate using magnetic particles conjugated to antibodies against the CD138 marker. The EasyStep Human CD138+ Positive Selection Kit II, Catalog #17877 (STEMCELL Technologies, Vancouver, BC, Canada) was used. Simultaneously, lymphocytes were isolated from blood samples by washing the cells 3–5 times with red blood cell lysis (RBC) buffer [86]. The CD138+ plasma cells and blood lymphocytes were used for genomic DNA isolation using the AllPrep DNA/RNA Micro Kit, Catalog #80284 (Qiagen, Hilden, Germany). Exome sequencing of peripheral blood lymphocytes and CD138+ bone marrow plasma cells was performed on the Illumina 4000 NGS platform. The Human All Exon version V6+UTR V6/SSELXT Human All Exon V6+UTR V6 enrichment panel Part #5190-8881, (Agilent Technologies, Santa Clara, CA, USA) was used to prepare the extended exome libraries for 9 patients, and the Illumina Truseq Exome kit, Catalog #20020614 (Illumina, San Diego, CA, USA) was used to sequence the exomes of two patients S7 and S12.

### 4.3. Data Processing and Somatic Variant Calling

The assessment of read quality was carried out with FastQC to calculate and visualize sequence quality metrics of raw and filtered reads [96]. The quality metrics were combined using MultiQC [97]. AfterQC (v0.9.7) or Bbduk (v39.01) were used to trim technical sequences and bases with a quality score lower than 20 Phred [98,99]. The paired-end reads passing processing of tumor and normal samples were aligned to the GRCh38 reference genome using BWA-MEM (v0.7.17) [100]. The bam files were processed using Picard MarkDuplicates (v2.26.11) to mark duplicates and improve the accuracy of downstream analysis (https://broadinstitute.github.io/picard/ accessed on 2 March 2022). Alignment quality was improved using the GATK (v4.2.5.0) quality score recalibration step with known sites dbsnp155 [101]. The quality metrics for alignment were collected by samtools stats, picard CollectAlignmentSummaryMetrics, ValidateSamFile, CollectInsertSizeMetrics, and deepTools plotCoverage [102,103]. The resulting bam files were then used for germline and short somatic variant calling in tumor-normal pairs for each sample. Germline SNPs and indels were called by GATK HaplotypeCaller with GVCF parameter, followed by merging GVCF by CombineGVCFs and performing joint genotyping by GenotypeGVCFs. Subsequently, the variant call set underwent filtration steps based on variant quality score recalibration. Further refinement was achieved by applying filters, including a read depth (DP) threshold greater than 10 and an alternative allele depth (AD) threshold of greater than 5 using bcftools (v1.18). CoMut was used for SNP visualization [64]. Multiple bioinformatic tools, including Mutect2 (v4.2.5.0), Strelka2 (v2.9.10) [104], VarScan2 (v2.4.4) [105], and Somaticsniper [106], identified somatic variants. SNVs and INDELs were detected by at least two of the callers. Mutect2, VarScan2, Strelka2, or Somaticsniper were combined using SomaticSeq (v3.7.3) [107]. Variants were annotated using Ensembl Variant Effect Predictor (VEP) (v110) [108].

### 4.4. Mutational Signatures

Mutational signature analysis was performed using SigProfilerAssignment [54].

### 4.5. Identification of G4-Forming Motifs around the Somatic Variants

Putative G-quadruplex sequences in the multiple myeloma exome were computationally defined as stretches of at least four (G)n runs separated by variable sequence loops of up to 10 nucleotides each:

In this study we defined two main categories of G-quadruplexes and used the following expressions using the Python re module to work with regular expressions:

(1) Weak G4 consisting of stretches of two consecutive guanines:

“G{2}\D{1,10}G{2}\D{1,10}G{2}\D{1,10}G{2}”/“C{2}\D{1,10}C{2}\D{1,10}C{2}\D{1,10}C{2}”

(2) Strong G4 consisting of stretches of three to four consecutive guanines:

“G{3,4}\D{1,10}G{3,4}\D{1,10}G{3,4}\D{1,10}G{3,4}”/“C{3,4}\D{1,10}C{3,4}\D{1,10}C{3,4}\D{1,10}C{3,4}”

The corresponding motifs were searched for in sequences around the points for somatic mutation sites: the analyzed sequence included 70 nucleotides up- and downstream from the detected somatic alterations including SNPs and indels.

Prediction of the G4-forming properties were carried out on sequences extracted from the reference human genome GRCh38 and information about the germinal variants carried by patient in these intervals was applied to the corresponding sequences (see Supplementary Materials Table S4). The same type of analysis was performed for prediction of G4-forming properties upon introduction of somatic mutations.

### 4.6. Statistical Evaluation

The chi-square test of independence was used to compare G4 strong context enrichment around mutation sites in tumors and randomly sampled regions. The contingency tables, chi-square statistic and *p*-value were obtained with scipy.stats.chi2 (scipy.stats.chi2_contingency) in Python 3.11.7. Similar procedure was performed for the comparison of the G4 strong plus G4 weak group against no G4 group.

Moreover, we employed the two-proportion z-test to determine a statistically significant difference between the proportions of G4 context occurrence near mutation sites in tumors and in randomly sampled sequences [109]. The z-scores and *p*-values were calculated with the proportions z-test function from the statsmodels.stats.proportion module in Python. The confidence interval for a proportion was calculated using the Wilson score method [110].

R packages ggplot2 and factoextra and Python libraries matplotlib and seaborn were used for data visualization.

## 5. Conclusions

MM is a highly heterogeneous disease that can vary widely among patients in terms of clinical manifestation, genetic characteristics, and response to treatment. This heterogeneity poses a challenge to the diagnosis and treatment of MM, as it can affect the prognosis and outcomes of individual patients. Understanding the biological and genetic factors that contribute to the development and progression of MM is critical to developing more targeted and effective treatments for this complex disease. Advances in research have already led to the identification of several distinct factors that determine the genetic heterogeneity of different MM subtypes with different molecular profiles. In this paper, we present the results of a study that identified another factor that contributes to the destabilization of the genetic material in MM, at least in some patients. We have shown that somatic mutations in regions of the genome that are predicted to form G4 structures are more frequent in tumor plasma cells in a fraction of patients. Thus, we have described another level of MM heterogeneity that may be linked to specific mutational signatures and mutational processes undergoing in different tumors. Further studies are needed to identify specific factors (most likely proteins involved in DNA metabolism—helicases, DNA polymerases, repair factors) that are directly involved in the generation of substitutions and other mutations in difficult-to-replicate regions of the genome enriched in the G4 context.

## Figures and Tables

**Table 1:** Characteristics of patients with multiple myeloma.

Patient	Sex	Age	Paraprotein	R-ISS	Performance Status
S7	M	81	IgG k	I	ECOG II
S12	F	61	IgG λ	II	ECOG II
P1	F	74	IgG k	II	ECOG II
P14	F	58	IgA k	I	ECOG I
P20	M	74	IgG k	III	ECOG III
P22	F	71	IgA λ	I	ECOG I
P23	F	73	IgG k	I	ECOG I
P30	M	69	IgG k	III	ECOG II
P34	M	83	IgG k	II	ECOG II
P37	M	64	IgA λ	II	ECOG III
P48	F	56	IgG k	III	ECOG III

**Table 2:** Number of somatic mutations and G4 context enrichment around points of somatic mutations in analyzed patients.

Patient	Number of Somatic Mutations Identified in Tumor	G4 Enrichment in the Regions of Somatic Mutations
S7	115	No
S12	63	G4 strong
P1	87	No
P14	234	No
P20	182	No
P22	191	No
P23	119	G4 strong
P30	662	No
P34	267	No
P37	82	G4 strong
P48	227	G4 weak + G4 strong ^$^

^$^ No significant enrichment in the G4 strong context; however, this sample carried a high percentage of mutations in the G4 weak context, and significance was observed for the combined group (G4 strong plus G4 weak) when compared to the same group in randomly generated set.

## Data Availability

Data are contained within the article and Supplementary Materials.

## Supplementary Materials

The following supporting information can be downloaded at: https://www.mdpi.com/article/10.3390/ijms25105269/s1.
