# Supplementary material for: G-Quadruplex Forming DNA Sequence Context Is Enriched around Points of Somatic Mutations in a Subset of Multiple Myeloma Patients
Source: Int J Mol Sci. 2024 May 12;25(10):5269. doi: 10.3390/ijms25105269 (PMC11121618; doi:10.3390/ijms25105269)
Supplement: Supplementary file 1 [file ijms-25-05269-s001.zip › Supplementary_information.pdf]

Supplementary Information

Supplementary figures

Figure S1. Heatmap of the influence of somatic mutations on G4 prediction. Structures predicted in the region were classified as described in the Materials and Methods section, using sequence information from the reference genome and data from germinal mutation calling. Somatic mutations altered the predicted structure of the region in 33 cases.

Figure S2. Distribution of genetic variant consequences associated with G4 prediction changes due to somatic Mutations. The pie chart categorizes and visualizes the frequency of genetic variant consequences in cases where the presence of somatic mutations led to changes in G4 prediction. Changes in G4 were predicted in the 3' and 5' untranslated regions (UTRs) of genes, as well as in introns, exons and intergenic regions. Each pie segment proportionally represents the count of each variant type.

Figure S3. Germinal variants with high or moderate impact found in the genes encoding DNA repair factors found in patients. Variants with high or moderate significance in the genes affecting various DNA repair processes were plotted for each of the 11 patients. CoMut was used for SNPs visualization [1].

1. Crowdis, J.; He, M.X.; Reardon, B.; van Allen, E.M. CoMut: Visualizing Integrated Molecular Information with Comutation Plots. *Bioinformatics* **2020**, *36*, 4348–4349. doi:10.1093/BIOINFORMATICS/BTAA554.

Supplementary tables

Table S1. Common rsID found in patients S12, P23, P37, which were absent in other patients.

rsID	GnomAD_AF	Gene	Gene function	Consequence
rs11250255 (minor allele T)	0.04507 (*)	*WDR37*	WD repeat domain 37	splice_polypyrimidine_tract_ variant&intron_variant
rs59626794	0.08812 (*)	*TSKS*	testis specific serine kinase substrate	inframe_deletion (repeats)
rs10999212	0.2211	*NPFFR1*	neuropeptide FF receptor 1	synonymous_variant
rs12130207	0.1829	*LRRC8B*	leucine rich repeat containing 8 VRAC subunit B	synonymous_variant
rs2297595	0.08532 (*)	*DPYD*	dihydropyrimidine dehydrogenase	missense_variant
rs76007555	0.05492 (*)	*TTC23*	tetratricopeptide repeat domain 23	synonymous_variant
rs10860577	0.1354	*SCYL2*	SCY1 like pseudokinase 2	synonymous_variant
rs17855473	0.1656	*AGFG2*	ArfGAP with FG repeats 2	missense_variant
rs17803441	0.04905 (*)	*SLC25A32*	solute carrier family 25 member 32	missense_variant
rs3098238	0.0503 (*)	*DCAF13*	DDB1 and CUL4 associated factor 13	synonymous_variant
rs79383654	0.06025 (*)	*LARP7*	La ribonucleoprotein 7, transcriptional regulator	missense_variant
rs2076165	0.1413	*TENM1*	teneurin transmembrane protein 1	synonymous_variant
rs3781413	0.1421	*CTBP2*	C-terminal binding protein 2	synonymous_variant
rs372118432	0.1 (AF)	*CTBP2*		inframe_insertion
rs532090538	0.0204 (*)	*PCDH12*	protocadherin 12	inframe_insertion

(*) – denote rsID with allele frequency below 0.1 that were discussed in the study

Table S2. Molecular function of genes carrying the rsID from Table S1 with AF < 0.1. The information was obtained from GeneCards database (genecards.org), assessed on December 1, 2023

Gene	Function
WDR37	*WDR37* (WD Repeat Domain 37) is a Protein Coding gene. Diseases associated with *WDR37* include Neurooculocardiogenitourinary Syndrome and Coloboma Of Macula.
TSKS	This gene may play a role in testicular physiology, spermatogenesis or spermiogenesis. Expression of the encoded protein is highest in the testis and downregulated in testicular cancer. Associated with Testicular Cancer.
DPYD	The protein encoded by this gene is a pyrimidine catabolic enzyme and the initial and rate-limiting factor in the pathway of uracil and thymidine catabolism. Mutations in this gene result in dihydropyrimidine dehydrogenase deficiency, an error in pyrimidine metabolism associated with thymine-uraciluria and an increased risk of toxicity in cancer patients receiving 5-fluorouracil chemotherapy. Diseases associated with *DPYD* include Dihydropyrimidine Dehydrogenase Deficiency and Bunion.
TTC23	*TTC23* (Tetratricopeptide Repeat Domain 23) is a Protein Coding gene. Predicted to be involved in positive regulation of smoothened signaling pathway. Diseases associated with *TTC23* include Chromosome 15Q26-Qter Deletion Syndrome and Kbg Syndrome.
SLC25A32	This gene encodes a member of the P(I/L)W subfamily of mitochondrial carrier family transport proteins. The encoded protein transports folate across the inner mitochondrial membrane. *SLC25A32* (Solute Carrier Family 25 Member 32) is a Protein Coding gene. Diseases associated with *SLC25A32* include Exercise Intolerance, Riboflavin-Responsive and Multiple Acyl-Coa Dehydrogenase Deficiency, Mild Type. Among its related pathways are Metabolism of water-soluble vitamins and cofactors and Metabolism.
DCAF13	*DCAF13* (DDB1 And CUL4 Associated Factor 13) is a Protein Coding gene. Enables estrogen receptor binding activity. Predicted to be involved in maturation of SSU-rRNA from tricistronic rRNA transcript (SSU-rRNA,

	5.8S rRNA, LSU-rRNA). Located in several cellular components, including centrosome; cytosol; and nuclear lumen. Part of Cul4-RING E3 ubiquitin ligase complex. Part of the small subunit (SSU) processome, first precursor of the small eukaryotic ribosomal subunit.
LARP7	*LARP7* (La Ribonucleoprotein 7, Transcriptional Regulator) is a Protein Coding gene. Diseases associated with LARP7 include Alazami Syndrome and Alazami-Yuan Syndrome. This gene encodes a protein which is found in the 7SK snRNP (small nuclear ribonucleoprotein). This snRNP complex inhibits a cyclin-dependent kinase, positive transcription elongation factor b, which is required for paused RNA polymerase II at a promoter to begin transcription elongation. RNA-binding protein that specifically binds distinct small nuclear RNA (snRNAs) and regulates their processing and function.

Table S3. SNPs affecting *DDX5* gene that are absent in the group without G4 strong enrichment.

Patient	*DDX5* polymorphisms detected	Position	Consequence	GnomAD AF
P23	rs62073204	chr17:64501194	(G->C) intron variant<br>MIR5047: 500B Downstream Variant	0.006
P37	rs1427463	chr17:64496464	*DDX5* downstream variant<br>*POLG2*: Missense Variant	0.29
	rs2075552	chr17:64504635	C->T intron variant	0.14
	rs16947825	chr17:64505738	C-G intron variant	0.13
	rs2075551	chr17:64505810	C->G intron variant	0.13
	rs2075550	chr17:64505849	A->G intron variant	0.28
	rs2075549	chr17:64505893	C->T intron variant	0.05
	rs117604948	chr17:64506696	G->A upstream variant	0.05
S12	rs16947825	chr17:64505738	(C->G) intron variant	0.13
	rs2075551	chr17:64505810	(C->G) intron (1) variant	0.12
