# Supplementary figures and images for: G-Quadruplex Forming DNA Sequence Context Is Enriched around Points of Somatic Mutations in a Subset of Multiple Myeloma Patients
Source: Int J Mol Sci. 2024 May 12;25(10):5269. doi: 10.3390/ijms25105269 (PMC11121618; doi:10.3390/ijms25105269)

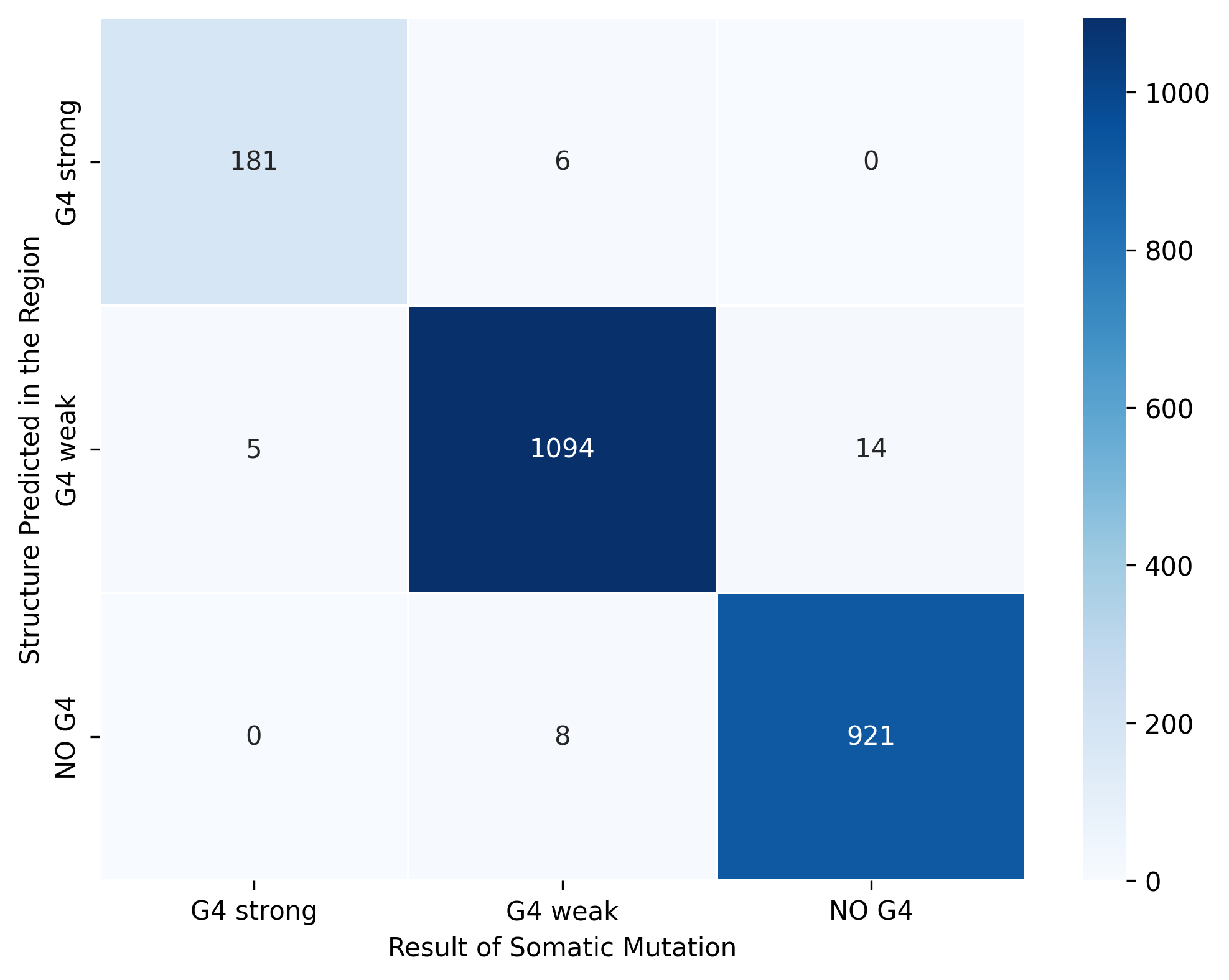

Supplement: Supplementary file 1 [file ijms-25-05269-s001.zip › Figure_S1.png]

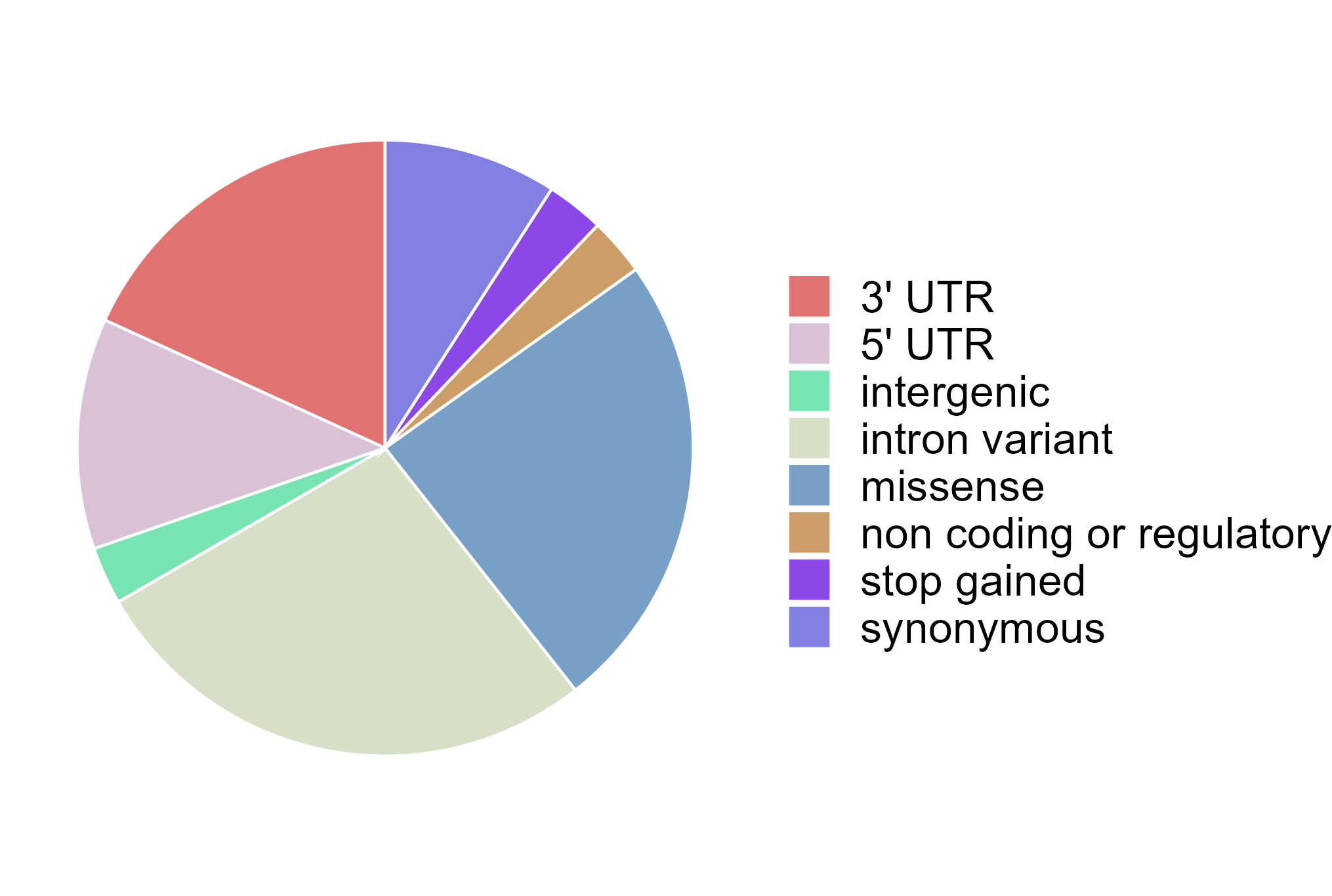

Supplement: Supplementary file 1 [file ijms-25-05269-s001.zip › Figure_S2.png]
